# Prenatal stress and child externalizing behavior: effects of maternal perceived stress and cortisol are moderated by child sex

**DOI:** 10.1186/s13034-023-00639-2

**Published:** 2023-08-07

**Authors:** Leonie Fleck, Anna Fuchs, Silvano Sele, Eva Moehler, Julian Koenig, Franz Resch, Michael Kaess

**Affiliations:** 1 Department of Child and Adolescent Psychiatry, Centre for Psychosocial Medicine, University Hospital Heidelberg, Heidelberg, Germany; 2 Department of Child and Adolescent Psychiatry, Saarland University Medical Center, Homburg, Germany; 3 Department of Child and Adolescent Psychiatry, Psychosomatics and Psychotherapy, University of Cologne, Faculty of Medicine and University Hospital Cologne, Cologne, Germany; 4 University Hospital of Child and Adolescent Psychiatry and Psychotherapy, University of Bern, Bern, Switzerland

**Keywords:** Externalizing behavior, Prenatal Stress, Cortisol, Sex differences

## Abstract

**Background:**

Externalizing behavior problems are related to social maladjustment. Evidence indicates associations between prenatal stress and child behavioral outcomes. It remains unclear how psychological distress vs. biological correlates of stress (cortisol) differentially predict externalizing behavior, and how their effects might differ as a function of child sex.

**Method:**

108 pregnant women from the community collected salivary cortisol and reported their perceived stress during each trimester of pregnancy. At child age 9 years (*M* = 9.01, *SD* = 0.55), 70 mothers and children reported on child behavior. Structural equation modelling was used to analyze how cortisol levels and perceived stress during pregnancy predicted current child externalizing behavior, considering the moderating effect of child sex.

**Results:**

Perceived stress predicted higher externalizing behavior in boys (*β* = 0.42, *p* = 0.009) and lower externalizing behavior in girls (*β* = − 0.56, *p* = 0.014). Cortisol predicted lower externalizing behavior in boys (*β* = − 0.81, *p* < .001) and was not related to girls’ externalizing behavior (*β* = 0.37, *p* = 0.200).

**Discussion/Conclusion:**

Prenatal stress affected externalizing behavior differently in girls vs. boys. These response patters in turn differed for indicators of psychological vs. biological maternal stress, encouraging an integrated approach. Findings indicate that perceived stress and cortisol may affect child development via different trajectories.

**Supplementary Information:**

The online version contains supplementary material available at 10.1186/s13034-023-00639-2.

## Introduction

Externalizing behavior comprises behavioral problems that are displayed in the child’s outward behavior with their external environment [1]. Behaviors incorporated under the term are often characterized by aggression and defiance, but also behaviors that are not inherently anti-social, such as hyperactivity and impulsivity [1]. Clinically, children who exhibit more externalizing behavior tendencies are at greater risk for developing mental disorders such as antisocial personality disorder, attention deficit hyperactivity disorder (ADHD), and substance use disorders [2, 3]. Socially, externalizing behaviors predict delinquent behavior and overall academic and psychosocial maladjustment [4–6], which emphasizes the need for empirical and clinical attention. The identification of risk factors and developmental pathways toward externalizing behavior can help identifying targets for prevention.

Certain temperamental traits are associated with a higher likelihood of externalizing behavior. Novelty seeking for example as a temperamental characteristic describes a behavioral tendency towards impulsivity and rule-breaking, and may therefore overlap with or promote externalizing behavior. Indeed, externalizing behavior and disruptive disorders have been shown to be associated with novelty seeking [7, 8] and there is empirical evidence for a strong association between novelty seeking and the externalizing disorder of ADHD [9]. Temperamental disinhibition may also account for the co-occurrence of externalizing problems [10–12]. Two different theories are aiming at an explanation for the relationship between certain temperamental styles and psychopathology: According to a spectrum perspective, psychopathology and temperament run along the same continuum, with psychopathology marking an extreme [10]. According to a vulnerability perspective, temperamental style (in combination with environmental adversity) is seen as more conceptually different, but increases the risk for the development of certain types of psychopathology [10]. Examinations of the meta-structure of psychopathology stress the relevance of trait impulsivity and disinhibition for psychopathology of the externalizing spectrum [13, 14]. Temperamental response tendencies such as novelty seeking and impulsiveness could serve well as dimensional indicators of or risk factors for the externalizing spectrum in community samples, where pathological degrees of externalizing behavior problems might only be present in 5% of children [15].

### Prenatal stress: methodology and associations with externalizing behavior

According to the biosocial interactional model, prenatal adversity such as maternal smoking, malnutrition or maternal stress may contribute to the development of externalizing behaviors [1]. Prenatal stress has also been linked to difficult temperament and more general child behavior problems [16, 17]. Maternal prenatal stress is commonly examined by focusing on two different components, *psychological* stress and *biological* correlates of stress [16]. *Psychological* stress during pregnancy is often measured by assessing stressful life events, aspects of psychological symptomatology, anxiety and depression or by assessing subjectively *perceived stress*, i.e. the emotional response and perceived ability to meet the situational demands [18]. While the measurement of actual events is often considered to be of greater objective value, recall bias could impact associations between exposure and outcome [19]. Moreover, it may be the individual’s perceptions and evaluations of occurring events that are causing stress rather than the event itself [16, 20]. Thus, for studies examining adverse outcomes of stress during pregnancy, the assessment of perceived stress may be of great interest.

The hypothalamic–pituitary–adrenal-axis (HPA-axis) is a complex neuroendocrine system regulating the body’s stress response. The release of the hormone cortisol depends on the negative feedback loop of the HPA axis. In response to stressors, increased release of cortisol elevates the availability of glucose and facilitates the capability of a response to situational challenges [21]. Cortisol is therefore often studied as a *biological* correlate of stress in women during pregnancy. In pregnant women, elevated maternal cortisol levels can affect the development of important physiological regulatory systems in the fetus such as HPA-functioning, the limbic system or the prefrontal cortex [17]. “Fetal programming” has been suggested as one of the biological mechanism connecting prenatal stress and offspring development [22–25]. Biological mechanisms that have been suggested include, i.e., influences of prenatal stress on synaptic development, epigenetic changes, or influences on brain network connectivity [17]. For example, maternal cortisol during pregnancy might influence offspring stress reactivity by altering glucocorticoid receptor density in the offspring [25].

In several studies associations between maternal psychological prenatal stress and child externalizing problems have been investigated. Studies infancy and toddlerhood have focused on temperamental outcomes such as higher distress to limitations and disruptive temperament, showing significant effects of perceived stress in infancy [26, 27], but a reverse effect in the same sample in toddlerhood [28]. Perceived stress was however related to more externalizing problems as measured by the Childhood Behavior Checklist (CBCL) in toddlerhood [28]. In studies with follow-ups in later childhood and early adolescence, the CBCL [29, 30], Strengths and Difficulties Questionnaire [31], or diagnoses of behavioral disorders such as conduct disorder and oppositional defiant disorder based on the Diagnostic and Statistical Manual [32, 33] were used to reflect externalizing difficulties. Prenatal depressive symptoms [32], stressful live events [29] and perceived stress [33] were predictors of externalizing behavior. However, one study using a Nicaraguan higher risk sample did not find significant associations between prenatal maternal distress and child psychiatric problems, including externalizing problems [30].

Fewer studies have investigated the link between maternal cortisol during pregnancy and later child externalizing behaviour problems, producing inconsistent results. One study found a positive link between morning cortisol levels in pregnant women and total as well as externalizing behaviour problems at child age nine [30], while another study failed to show such association in two-year-olds [28]. In a third study it was expected that maternal plasma cortisol and amniotic cortisol would predict child distress to limitations via lower birth weight [26], but neither a main effect of maternal cortisol on child temperament nor the indirect effect via birth weight were significant. Thus, findings examining the association between prenatal maternal cortisol and child externalizing behaviour outcomes are clearly sparser and less conclusive than evidence on the effects of psychological prenatal stress.

Interestingly, a review concluded that the two components studied as indicators of prenatal stress, i.e. psychological stress and cortisol, often only show weak associations or none at all [34]. There are only a few studies that investigated a link between prenatal stress and child externalizing behavior problems including both subjective and biological stress pathways, challenging the direct comparison of both effects. The few studies that included both risk factors mostly reported significant effects of either one or the other [26, 28, 30]. One possible explanation is that subjective maternal stress and cortisol levels influence child development via different trajectories. Consequently, more research integrating both psychological and biological measures is called for [16], especially with regards to externalizing behavior as a child outcome.

### Sex-dependent effects of prenatal stress

Overall, if studies examine a potential role of child sex in the association between prenatal psychological stress and child externalizing behavior, most studies indeed do report significant differences in boys and girls [35]. For example, boys were found to be more vulnerable to develop increased general emotional-behavioral problems, more externalizing problems such as ADHD symptoms, conduct disorder symptoms, and a more irritable temperament in response to prenatal maternal psychological stress or psychopathology [36–39]. In contrast, pre- and postnatal maternal anxiety trajectories were found to even lower the risk for conduct disorder in girls, while again they increased the risk in boys [40]. Concurrently, there is also evidence either pointing at a higher vulnerability in girls or failing to support any sex differences. For example, girls seemed to develop more externalizing symptoms in response to maternal prenatal depressive symptoms [41], whereas no differences in boys’ and girls’ responses to maternal stressful life events during early pregnancy were reported by [31]. In conclusion, even though there are some inconsistencies in results highlighting sex differences in the association between prenatal psychological stress and externalizing behavior, there is a trend towards higher vulnerability in boys.

In line with literature on prenatal psychological stress, for prenatal biological stress, the literature overall points to adverse outcomes of elevated cortisol during pregnancy. However, some studies investigating sex interaction effects reported opposite effects depending on sex: Whereas higher cortisol predicted more emotional reactivity in girls, it predicted less emotional negativity [42] and less emotional reactivity [43] in boys. Another study found lower callous-emotional traits in girls exposed to higher prenatal cortisol, but no effect for boys [44]. Overall, while studies investigating sex-dependent effects of prenatal maternal cortisol are still too sparse to identify a comprehensive pattern, the ones that exist relatively consistently report sex differences in response to prenatal stress. A failure to include sex as a moderator in studies of prenatal stress and child outcome may thus lead to meaningful associations being overlooked [35, 45]. In conclusion, more research is needed in order to dismantle the sex-dependent patterns of vulnerability to prenatal stress.

### Present study

The aim of the current study was to investigate the effects of both maternal perceived stress and cortisol levels during pregnancy on child externalizing behaviour at 9 years of age in a community sample. In order to map externalizing behaviour in a community sample, we considered conduct problems as well as temperamental tendencies such as impulsivity and novelty seeking as indicators. We hypothesized that (H1) higher maternal perceived stress and (H2) higher maternal cortisol levels would predict more child externalizing behaviour. As prior studies have suggested that outcomes of prenatal stress may be sex-specific, we aimed to investigate whether these effects differed in their strength or direction for girls vs. boys. We predicted that (H3) the association between maternal perceived stress and externalizing behaviour would be stronger for boys. Regarding maternal cortisol and sex-dependent effects on externalizing problems, research is still scarce, and we investigated this interaction effect in an explorative manner (E1). To our knowledge, the present study is the first to directly compare these possible moderating effects of sex between the influence of perceived stress and that of cortisol.

## Materials and methods

### Participants

Healthy, pregnant women with singleton pregnancies were recruited through cooperating gynecologist medical practices, local newspapers and websites. Mothers unable to understand the German language, in advanced pregnancy (> 19 weeks) or not able to participate in laboratory assessments five months after childbirth were not included into the study. One-hundred and eight mothers took part in the initial assessments during pregnancy (see [43, 44] for the course and interrelations of prenatal stress measures and infant development). Assessments took place during the first trimester (t1), second trimester (t2) and third trimester (t3). Infancy assessments took place at 4 months (t4) and 5 months (t5). At t6 (approximately nine years of age), 70 mothers and their children agreed to participate in the follow-up, resulting in a retention rate of 65% after ten years.

### Procedure

**Fig. 1:**
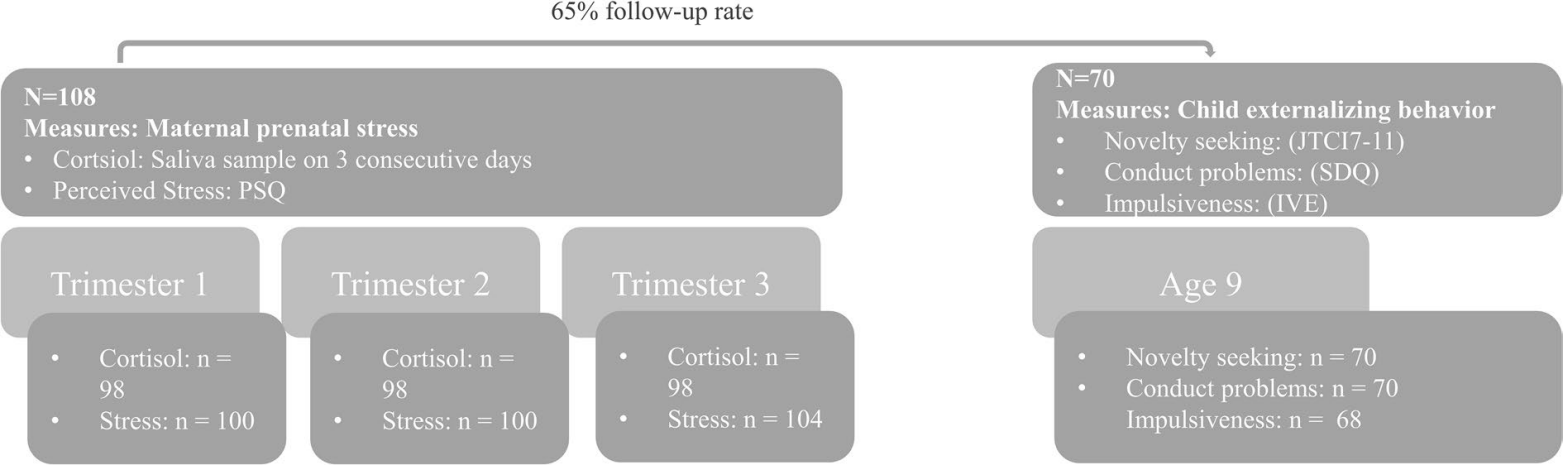
*Assessment Time Points and Study Variables. PSQ* Perceived Stress Questionnaire, *JTCI* Junior Temperament and Character Inventory, *SDQ* Strengths and Difficulties Questionnaire, *IVE* Impulsiveness-Venturesomeness-Empathy questionnaire

The study was approved by the Ethics Committee of the *Faculty of Medicine at the University of Heidelberg*. Through t1-t6, informed consent was obtained from all mothers. At t6 children were also asked to give their assent if they wanted to participate in the assessment. During each pregnancy trimester (t1-t3), both salivary cortisol and perceived stress were assessed. The appointment at t6 included the completion of questionnaires: Mothers reported on their child’s novelty seeking and conduct problems and children provided self-report on their impulsiveness (see Fig. 1).

### Measures

#### Stress

Biological and subjective measures of stress were assessed.

##### Biological stress

*Salivary cortisol* was measured using Sarstedt^®^ salivettes on three consecutive days in each trimester, resulting in 9 samples per participant. Participants were instructed to chew on the salivette for two minutes in a quiet and non-stressed situation at home within a time window from 11am to 1 pm. Individual cortisol samples have been shown to be predictive of the overall mean cortisol secretion throughout the day, with the highest predictive value at 6 h after awakening [48]. Samples were stored at − 20 °C. Cortisol concentrations were analyzed in the pharmacological laboratory of the University of Heidelberg. Samples were centrifuged for seven minutes at 3000 rpm. Intra- and interassay variation coefficients were below 6% and below 15% respectively. Per participant, 100 µl were analyzed in a specific in-house radioimmunoassay with a detection limit of 0.15 ng/100 ml.

##### Perceived stress

Mothers completed the 20-item version of the *Perceived Stress Questionnaire* [49, 50]. The PSQ assesses the subjective stress experience using the four scales “worries”, “tension”, “joy” and “demands” with five items each on a 4-point Likert scale ranging from “0—almost never” to “3—most of the time”. Example items include “You have too many things to do” and “You are full of energy”. Items of the “joy” scale are reversed before calculation of the total score. Higher scores indicate a higher degree of perceived stress. The PSQ has been established as a valid and economic instrument for stress research [50]. Total scores are divided by 3 and multiplied by 100 in order to display a possible scale range from 0 to 100. The PSQ total score was used in the current study. Internal consistency was α = 0.95.

#### Externalizing behavior

Three indicators of externalizing behavior were assessed:

##### Novelty seeking

At age 9 (t6), mothers evaluated their child’s temperament according to Cloninger’s psychobiological model using the 84-item *Junior Temperament and Character Inventory 7-11R* [51]. It measures the four temperament dimensions of harm avoidance, novelty seeking, perseverance and reward dependence and the three character dimensions self-direction, cooperation and self-transcendence. The temperament dimensions are assumed to be based on different underlying neurotransmitter systems and indicate individual differences in associative conditioning. Response categories on a 4-point Likert scale range from 0 (“no) to 4 (“yes”). The JTCI has established validity and its factor structure has been confirmed [51]. The novelty seeking scale (14 items, possible scale score range: 0 to 56; α = 0.81) was used for the purpose of this study. It comprises the subscales exploratory excitability (e.g. hard to stop when something has caught his/her curiosity), impulsiveness (e.g. acts according to his/her momentary affects, without thinking), extravagance (e.g. has intense emotional states (very happy, very angry)) and disorderliness (e.g. provokes and bothers others).

##### Conduct problems

Mothers also completed the parent version of the *Strengths and Difficulties Questionnaire* [52, 53]. Comprising 27 items, the SDQ is a brief screening instrument for child emotional and behavioral problems with good psychometric properties [54, 55]. Response categories on a 3-pont Likert scale range from 0 (“not true") to 2 (“certainly true”). In the present study, the conduct problems subscale was used (5 items, possible scale range: 0 to 10; α = 0.56) (e.g. often has temper tantrums or hot tempers; often lies or cheats).

##### Impulsiveness

Children completed the German version of the *Impulsiveness-Venturesomeness-Empathy* (IVE) questionnaire [56, 57]. The impulsiveness scale (16 items, possible scale range 0 to 16) was used in the current study and assesses cognitive and motivational aspect of impulsivity such as a lack of consideration of consequences of one’s own behavior, a focus on immediate rewards, and a lack of focus on future goals (e.g. I often get into trouble because I act without thinking; Sometimes I just don’t follow rules and regulations). Response options are 1 “yes” and 0 “no”. Findings showing that children scoring higher on impulsiveness make more commission errors (non-inhibited trials) on a Go-NoGo task support the validity of the scale [58]. Internal consistency of the impulsiveness scale in our sample was α = 0.78.

### Data analysis

#### Data collection

Across all three trimesters, 940 saliva samples were collected. Samples were excluded from the analyses if the sample had been taken before 11am or after 2 pm or if the determined cortisol level exceeded 10 nmol/l (7.8% of samples). Allowing samples taken up to one hour outside the requested time window to be included in analyses increased the number of usable samples from 815 (86.7%) to 876 (93.2%) and did not change the overall study results. Per trimester, the three cortisol samples sampled on three consecutive days were averaged for each mother, resulting in a trimester 1 average cortisol score, a trimester 2 average cortisol score, and a trimester 3 average cortisol score. Average scores were also calculated when single samples were missing or excluded. However, in some instances, no trimester average could be calculated as all three samples of a trimester were missing or excluded (trimester 1: *n* = 2, trimester 2: *n* = 2, trimester 3 = n = 3, all three trimesters: *n* = 2). *N* = 5 perceived stress reports (trimester 1: *n* = 1; trimester 2: *n* = 2; trimester 3: n = 2) and *n* = 2 IVE impulsiveness reports were missing.

#### Structural equation model

To build the comprehensive model, structural equation modelling (SEM) was used. Path coefficients represent beta values, i.e., they indicate the expected change in SD of the outcome given a predictor variable change of one SD. Beta coefficients can be interpreted as small effects size when < 0.20, moderate between 0.20 and 0.49, and large > 0.50 [60]. Confidence intervals provide information about the certainty of effects.

##### Structural model/main analyses

We modelled paths from the latent means of pregnancy cortisol and pregnancy perceived stress to predict the latent externalizing behavior variable.

##### Measurement model

SDQ conduct problems, JTCI novelty seeking and IVE impulsiveness were modelled to load on a latent externalizing behavior variable. Average cortisol scores from each trimester were modelled to load on a “latent mean” overall pregnancy cortisol variable. In order to reduce model complexity, loadings of all three assessments were set to 1 (unstandardized coefficient), and error variances were set to be equal for all three assessments. Equally, PSQ scores from each trimester were modelled to load on a latent mean of pregnancy perceived stress, with equal error variances and factor loading of 1 for all assessments.

##### Group factor

Child sex was included as a group factor, parameters were allowed to differ between groups. Wald tests were used to determine which parameters differed significantly between groups, allowing for different regression parameters between boys and girls of the latent cortisol and perceived stress variables on the latent externalizing behaviour. Significant group differences regarding the paths from the structural model indicate that there is a moderation effect of child sex. Additionally, we allowed for a different residual variance of the latent externalizing behaviour and for different intercepts in all the latent variables between boys and girls. The other parameters (measurement model intercepts and coefficients, error (co)variances) were constrained to be the same between boys and girls for interpretational reasons and to reduce model complexity.

**Fig. 2:**
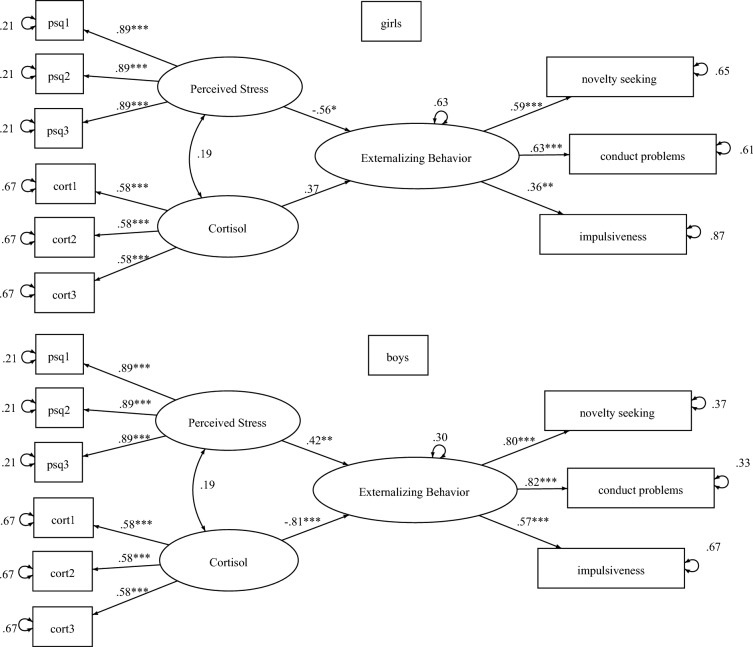
Estimated structural equation model of the relationship between maternal perceived stress and cortisol levels during pregnancy and child externalizing behavior at age 9. *Note.* *p < .05, ** *p* < .01, ***p < .001. Square boxes = observed variables. Ellipses = latent variables. Standardized coefficients. Error terms represent the not explained variance of variables (1—*R*^2^) in the standardized output. Effects of cortisol and perceived stress on externalizing behavior (structural coefficients), residual variance of the latent externalizing behavior, and intercepts of all the latent variables were allowed to vary between boys and girls. Other parameters were constrained to be the same between both groups. Constrained parameters can *appear* different for boys vs. girls because values were standardized within groups.

The structure of the SEM will also be visually depicted (Fig. 2/Results section).

##### Model fit

For SEM, a combination of fit indices is recommended in order to determine model fit. Model fit is deemed good at a *p*-value of chi square that is > 0.05. Root mean square error approximation (RMSEA) is considered acceptable at values < 0.07, good at values < 0.05 and excellent at values < 0.03. The comparative fit index (CFI) and Tucker-Lewis index (TLI) indicate acceptable model fit at values > 0.90 and good fit at values > 0.95 [59]. Relative model fit between two models (including vs. not including child sex as a group factor) can be evaluated based on Akaike’s information criterion (AIC), where the model with the smaller values of AIC is preferred.

Analyses were carried out using Stata 16. Level of significance was set at *p* < 0.05. Because of missing values and attrition, full information maximum likelihood estimation (FIML) was applied in order to obtain fitting parameters in the presence of missing values. Data on the grouping variable (child sex) is mandatory, leading to exclusion of *N* = 1 case of originally *N* = 108 due to missingness on the variable child sex. FIML estimates parameters based on all available information of the overall *N* = 107 dyads.

## Results

### Sample characteristics

During the pregnancy assessments, *N* = 107 mothers pregnant with *n* = 61 boys and *n* = 46 girls participated in the study. At the age 9 assessment (*M*_*age*_ = 9.01, *SD*_*age*_ = 0.55), mothers with *n* = 41 boys and *n* = 29 girls continued their participation. Figure 1 shows the *n* of each measurement at each assessment time point. Most mothers were still in a relationship with the child’s father (67.8%) and had obtained a university degree (62.9%). For a detailed description of sample characteristics, see Table 1. Mothers who dropped out of the study between the last pregnancy assessment and t6 did not differ from those who retained in the study with regards to perceived stress (*t*(106) = 0.442, *p* = 0.659), cortisol levels (*t*(104) = -0.184, *p* = 0.854), child sex (χ^2^(1) = 0.2015, *p* = 0.653), maternal partnership (χ^2^(3) = 6.9909, *p* = 0.072) or maternal education (χ^2^(6) = 7.0169, *p* = 0.319). Table 2 shows descriptive data of all important study variables in the full sample and in girls vs. boys. There were no significant sex differences regarding any of the study variables.

**Table 1:** Sociodemographic characteristics of the study sample at t6 (*N* = 70)

Demographics	*M* (*SD*; range)
*Mother age*	41.2 years (4.68; 27–51)
*Child age*	9.0 years (.55; 8–10)
	***n* (%)**
Mother partnership	
With child’s father	48 (68.8%)
With different partner	11 (15.7%)
No partnership	11 (15.7%)
Mother education	
General secondary school	2 (2.9%)
Intermediate secondary school	13 (18.6%)
University entrance diploma	11 (15.7%)
University degree	44 (62.9%)
Child sex	
Male	41 (58.6%)
Female	29 (41.4%)
Child school type	
Primary school	64 (91.4%)
Secondary school	6 (8.6%)

**Table 2:** Descriptive statistics of study variables

Variables	Full sample					Girls					Boys					Group diff	*p*
	*N*	*M*	*SD*	Min	Max	*N*	*M*	*SD*	Min	Max	*N*	*M*	*SD*	Min	Max	t	
cort1	103	2.17	1.27	0.267	7.15	45	2.15	1.22	0.26	5.33	57	2.21	1.32	0.3	7.15	− 0.25	0.802
cort2	98	3.13	1.62	0.7	8.3	45	3.21	1.80	0.7	8.3	52	3.05	1.47	0.7	6.9	0.48	0.631
cort3	98	3.43	1.56	0.267	7.57	45	3.59	1.69	0.37	7.57	52	3.30	1.45	0.27	6.55	0.92	0.361
psq1	107	41.21	20.66	5	88.33	46	41.49	21.32	6.67	88.33	60	41.24	20.42	5	81.67	0.06	0.952
psq2	100	37.60	20.28	0	86.67	44	37.92	18.81	0	81.67	55	37.52	21.69	3.33	86.67	0.10	0.922
psq3	104	39.89	21.77	1.667	98.33	45	39.79	21.54	1.67	86.67	58	39.91	22.32	6.67	98.33	− 0.03	0.977
Novelty seeking	70	26.39	7.71	11	53	29	25.45	6.54	14	39	41	27.05	8.46	11	53	− 0.85	0.362
Conduct problems	70	1.56	1.43	0	6	29	1.48	1.24	0	5	41	1.61	1.56	0	6	− 0.36	0.717
Impulsiveness	68	6.46	3.46	0	14	27	6.18	2.96	1	14	41	6.63	3.77	0	13	− 0.52	0.604

PSQ = Perceived Stress Questionnaire. Cort = Salivary cortisol. 1 = first trimester; 2 = second trimester, 3 = third trimester

### Preliminary analyses

Mean perceived stress and mean cortisol during pregnancy were not significantly correlated (*r* = 0.11, *p* = 0.245). For correlations among study variables in the full sample and girls and boys separately, see Table 3, 4, 5. Mean perceived stress was not associated with sociodemographic characteristics such as child sex (t(105) = 0.046, *p* = 0.964), partnership status (*F*(3104) = 0.78, *p* = 0.508) or job status (*F*(6,100) = 0.85, *p* = 0.532) reported during the first trimester of pregnancy. Likewise, mean cortisol was not associated with child sex (*t*(103) = 0.715, *p* = 0.476), partnership status (*F*(3102) = 1.78, *p* = 0.166) or job status (*F*(6,98) = 0.615, *p* = 0.718) reported during the first trimester of pregnancy. During pregnancy, mothers self-reported exposure to different kinds of medical risk factors: pregnancy complications were reported by 34% in trimester 1, 34% in trimester 2, 38% in trimester 3; alcohol consumption was reported by 6% in trimester 1, 2% in trimester 2, 5% in trimester 3; hospitalizations were reported by 9% in trimester 1, 14% in trimester 2, 26% in trimester 3. The presence of pregnancy complications, alcohol consumption, hospitalizations, or extant of maternal smoking during any trimester did not significantly relate to mean perceived stress or cortisol (all *p* > 0.05, see Additional file 1: Tables S1 and S2).

**Table 3:** Pairwise correlations full sample (Pearson’s r)

Variables	(1)	(2)	(3)	(4)	(5)	(6)	(7)	(8)	(9)
(1) cort1	1.000								
(2) cort2	**0.260**	1.000							
	**(0.011)**								
(3) cort3	**0.283**	**0.455**	1.000						
	**(0.005)**	**(0.000)**							
(4) psq1	0.192	0.019	− 0.063	1.000					
	(0.053)	(0.853)	(0.538)						
(5) psq2	0.147	0.183	0.064	**0.800**	1.000				
	(0.156)	(0.074)	(0.543)	**(0.000)**					
(6) psq3	**0.218**	0.147	0.076	**0.735**	**0.814**	1.000			
	**(0.029)**	(0.154)	(0.460)	**(0.000)**	**(0.000)**				
(7) Novelty seeking	− **0.307**	− **0.112**	− 0.062	0.215	**0.329**	0.143	1.000		
	**(0.011)**	**(0.371)**	(0.625)	(0.073)	**(0.006)**	(0.241)			
(8) Conduct problems	− **0.308**	− 0.126	− 0.094	0.040	− 0.014	− 0.120	**0.549**	1.000	
	**(0.011)**	(0.313)	(0.455)	(0.742)	(0.911)	(0.327)	**(0.000)**		
(9) Impulsiveness	− 0.224	0.044	− 0.032	0.081	0.080	− 0.054	**0.411**	**0.437**	1.000
	(0.073)	(0.732)	(0.803)	(0.513)	(0.527)	(0.666)	**(0.000)**	**(0.000)**	

*PSQ*  Perceived Stress Questionnaire, *Cort* Salivary cortisol, *1* first trimester, *2* second trimester, *3* third trimester. Values in brackets present *p* values. Correlations significant at a level of *p* < .05 are printed in bold

**Table 4:** Pairwise correlations girls (Pearson’s r)

Variables	(1)	(2)	(3)	(4)	(5)	(6)	(7)	(8)	(9)
(1) cort1	1.000								
(2) cort2	0.228	1.000							
	(0.136)								
(3) cort3	0.280	**0.484**	1.000						
	(0.065)	**(0.001)**							
(4) psq1	**0.392**	− 0.044	− 0.131	1.000					
	**(0.008)**	(0.775)	(0.391)						
(5) psq2	**0.311**	0.203	0.050	**0.837**	1.000				
	**(0.042)**	(0.186)	(0.748)	**(0.000)**					
(6) psq3	**0.297**	0.096	− 0.048	**0.774**	**0.810**	1.000			
	**(0.050)**	(0.534)	(0.757)	**(0.000)**	**(0.000)**				
(7) Novelty seeking	0.153	− 0.132	0.315	− 0.115	− 0.029	− 0.152	1.000		
	(0.436)	(0.496)	(0.096)	(0.552)	(0.883)	(0.441)			
(8) Conduct problems	0.097	− 0.074	0.325	− **0.478**	− **0.422**	− **0.436**	0.271	1.000	
	(0.625)	(0.703)	(0.085)	**(0.009)**	**(0.025)**	**(0.020)**	(0.155)		
(9) Impulsiveness	− 0.131	− 0.034	0.174	− 0.140	− 0.071	− 0.126	0.140	**0.384**	1.000
	(0.524)	(0.865)	(0.386)	(0.487)	(0.730)	(0.540)	(0.486)	**(0.048)**	

*PSQ*  Perceived Stress Questionnaire, *Cort*  Salivary cortisol, *1* first trimester, *2* second trimester, *3* third trimester. Values in brackets present *p* values. Correlations significant at a level of *p* < .05 are printed in bold

**Table 5:** Pairwise correlations boys (Pearson’s r)

Variables	(1)	(2)	(3)	(4)	(5)	(6)	(7)	(8)	(9)
(1) cort1	1.000								
(2) cort2	**0.312**	1.000							
	**(0.027)**								
(3) cort3	**0.289**	**0.422**	1.000						
	**(0.040)**	**(0.003)**							
(4) psq1	0.024	0.097	0.003	1.000					
	(0.863)	(0.500)	(0.985)						
(5) psq2	0.029	0.178	0.076	**0.778**	1.000				
	(0.839)	(0.212)	(0.608)	**(0.000)**					
(6) psq3	0.164	0.204	0.212	**0.711**	**0.821**	1.000			
	(0.231)	(0.152)	(0.136)	**(0.000)**	**(0.000)**				
(7) Novelty seeking	− **0.542**	-0.113	**-0.344**	**0.401**	**0.504**	0.293	1.000		
	**(0.000)**	(0.507)	**(0.040)**	**(0.009)**	**(0.001)**	(0.063)			
(8) Conduct problems	− **0.517**	− 0.168	**− 0.421**	**0.328**	0.189	0.038	**0.667**	1.000	
	**(0.001)**	(0.320)	**(0.011)**	**(0.036)**	(0.250)	(0.813)	**(0.000)**		
(9) Impulsiveness	− 0.272	0.086	− 0.173	0.201	0.145	− 0.020	**0.517**	**0.458**	1.000
	(0.094)	(0.613)	(0.314)	(0.209)	(0.378)	(0.901)	**(0.001)**	**(0.003)**	

*PSQ*  Perceived Stress Questionnaire, *Cort* Salivary cortisol, *1* first trimester, 2 second trimester, *3* third trimester. Values in brackets present *p* values. Correlations significant at a level of *p* < .05 are printed in bold

### Structural equation model

Both pregnancy cortisol and pregnancy perceived stress predicting externalizing behavior differed significantly for boys versus girls (cortisol: χ^2^(1) = 9.60, *p* = 002; PSQ: χ^2^(1) = 10.36, *p* = 0.001), suggesting a moderating effect of child sex for both predictors. In the structural model, latent mean pregnancy cortisol significantly negatively predicted externalizing behavior at age 9 for boys (*β* = -0.81, *p* < 0.001, 95% CI [− 1.15, − 0.46]) but we found no evidence for a relation to externalizing behavior in girls (*β* = 0.37, *p* = 0.200; 95% CI [− 0.20, 0.94]). Latent mean pregnancy perceived stress showed a positive association with externalizing behavior in boys (*β* = 0.42, *p* = 0.009, 95% CI [0.10, 0.74]) and a negative association in girls (*β* = − 0.56, *p* = 0.014, 95% CI [-1.01, -0.11]). For a visual depiction of the SEM, see Fig. 2.

In the measurement model, factor loadings of SDQ conduct problems (girls:* β* = 0.62, 95% CI [0.34–0.91]; boys:* β* = 0.82, 95% CI [0.67, 0.97]), JTCI novelty seeking (girls:* β* = 0.59, 95% CI [0.35, 0.83]; boys:* β* = 0.80, 95% CI [0.64, 0.95]) and IVE impulsivity (girls:* β* = 0.36, 95% CI [0.14, 0.57]; boys: *β* = 0.57, 95% CI [0.35, 0.79]) all were significant for the latent externalizing behavior variable (all *p* ≤ 0.001.). Thus, conduct problems and novelty seeking loaded similarly strong on latent externalizing behavior, whereas impulsiveness loaded still significantly but weaker on the latent factor. As indicated by error variances (1—R^2^), the latent externalizing behavior variable explained more variance in conduct problems, novelty seeking and impulsiveness in boys than it did for girls (see Fig. 2).

Comparison of AIC additionally supported selecting the model including child sex as a group variable (AIC = 465.52) over the model not considering child sex differences (AIC = 4698.10). For this final model, model fit was overall acceptable, as two fit indices were bordering the suggested cut-off (χ^2^(80) = 102.23, *p* = 0.048; RMSEA = 0.07; 90% CI [0.01–0.11]) and two incremental fit indices were reaching the cut-off for acceptable fit (CFI = 0.934; TLI = 0.942).

## Discussion

This study examined the longitudinal relationship between maternal stress during pregnancy and child externalizing behavior at age 9 years by assessing two components of maternal stress, i.e. subjective reports and maternal cortisol levels as indicators of biological stress. It further considered potential sex-specific differences in the associations between perceived stress, cortisol levels and child externalizing behavior. We found that higher maternal perceived stress during pregnancy was associated with decreased externalizing behavior in girls but increased externalizing behavior in boys. In contrast, higher pregnancy cortisol levels were linked to lower externalizing behavior in boys but there was no evidence for an effect of cortisol on externalizing behavior in girls. Our results are in line with prior studies reporting a stronger positive relationship between psychological maternal stress during pregnancy and externalizing behaviors for boys compared to girls, supporting our hypothesis regarding sex-specific effects of perceived stress [36, 39]. Moreover, our finding that the effect on girls’ externalizing behavior was not only weaker but actually directionally reversed is in line with results from [40], who found opposite effects of pre- and postnatal maternal anxiety trajectories for conduct problems in boy vs. girls. These results may be explained by different mechanisms during both pre- and postnatal periods.

First, it is important to note that our finding of higher maternal psychological stress dampening girls’ externalizing behavior problems does not rule out an unfavorable effect of stress on girls internalizing behavior problems. As has been proposed in recent reviews, prenatal stress may exacerbate sex-dependent vulnerabilities toward developing internalizing vs. externalizing problems [35, 61]. Male and female fetuses may be differently affected by challenges in the intrauterine environment. For example, studies have shown that the structure and connectivity of the amygdala are more strongly affected by prenatal stress in female fetuses, and that these neural alterations may be more specific to the development of fear and affective problems [35, 61]. Thus, whereas higher levels of maternal psychological stress may buffer girls from developing externalizing problems, they may still put them at risk for the development of internalizing problems.

Alternatively, a substantial number of women who report higher psychological stress during pregnancy continue to experience higher levels of stress after childbirth as well [40, 62]. It is possible that higher levels of perceived stress may have caused women to interact differently with their girls vs. boys postnatally compared to women with lower levels of perceived stress. For example, mothers with daughters high in negative emotionality seem to increase responsiveness when they report *higher* parenting stress, whereas mothers with sons high in negative emotionality seem to increase responsiveness when they report *lower* parenting stress [63]. These parenting patterns specific to the combination of maternal stress and child sex could possibly protect girls from the development of externalizing problems. In addition, girls and boys may respond differently to a mother who is more stressed or to environmental conditions that may be stressful for both mother and child. Some evidence suggests that stressful live events might be more strongly associated with externalizing and aggressive behavior in boys compared to girls [64, 65]. Differences in the response may be explained by sex-specific differences in the use of coping styles and socialization, with girls using more socially oriented coping techniques and boys leaning more towards impulsiveness, acting out and rule-breaking behaviour [66, 67]. Of note, confidence intervals of effects of perceived stress for both boys and girls ranged from small to large effect sizes, indicating a certain degree of uncertainty to the actual magnitude and interpretation of these results.

Regarding maternal cortisol, a different pattern emerged, such that for boys only there was a negative effect of prenatal maternal cortisol on externalizing behavior. Both the lower and the upper bound of the confidence interval of this association in boys indicated a moderate to strong effect. This finding of a protective effect of elevated cortisol levels during pregnancy in boys specifically mirrors the results of [43] and [68], who reported significant associations between higher pregnancy cortisol and easier temperament in boys. A possible explanation why cortisol could lead to more favorable outcomes may be that maternal cortisol shows a natural increase in the course of pregnancy. These increases benefit fetal organ and neurodevelopment and might prepare the onset of maternal behavior [69, 70]. In our low-risk study population, higher mean pregnancy cortisol levels may thus be an indicator of healthy development. Following this, future studies could investigate how different pregnancy cortisol trajectories relate to child mental health outcomes [71]. Also, it seems possible that lower mean pregnancy cortisol may have been indicative of attenuated maternal HPA axis functioning. Attenuated cortisol output is often found in clinical samples and populations characterized by higher levels of psychopathology, maltreatment or chronic stress [72, 73]. Interestingly, a profound number of studies has highlighted attenuated cortisol output in children and adolescents with externalizing behavior problems [74], with evidence suggesting that the association between lower cortisol and higher externalizing problems may be more pronounced in boys [75, 76]. It is hypothesized that those individuals may engage in risky behaviors in order to increase their physiological arousal [74]. Considering that maternal and fetal cortisol levels have been shown to correlate [77], and HPA activity has a genetic basis [78], mothers with hypocortisolism during pregnancy may transmit a predisposition of lower HPA (re)activity to their children, which in turn is associated with externalizing behavior especially in boys. Whether this hypothesis holds true needs to be investigated in future research.

Our results suggest that cortisol and psychological stress may influence child development via different trajectories. A large study (*N* = 3039 women from the community) has indicated that pregnancy cortisol levels are mainly influenced by biological and lifestyle factors (e.g. maternal age, parity, fetal sex, smoking, or sleep) rather than concurrent psychosocial stress [79]. Consequently, in addition to HPA-axis-functioning there may be other mechanisms accounting for the effect of psychological stress. Future studies are needed to investigate other potential mechanisms and physiological derivates of prenatal psychological stress.

Regarding the measurement model of externalizing behavior, it is evident that IVE impulsiveness had the lowest factor loading compared to the other two indicator variables, SDQ conduct problems and JTCI novelty seeking. I.e., effects of prenatal stress are less relevant to this indicator of externalizing behavior specifically. A lower factor loading could be the results of different informants (child self-report vs. maternal report) or might reflect that there are distinct subtypes of disinhibition and impulsiveness [80]. A number of neuroendocrine mechanisms have been hypothesized to play a role in early life stress and child outcome [81], and different mechanisms might be involved in the development of temperamental style vs. that of behavioral disorders. Moreover, whereas there is consistent evidence for associations between impulsiveness and novelty seeking with externalizing problems (strengthening vulnerability or spectrum theories of the association between temperament and psychopathology), it is of note that novelty seeking is not inherently pathological but also associated with positive outcomes such as creativity [82, 83]. Thus, the differentiation of antecedents and consequences of supposedly distinct types of impulsiveness remains an important objective.

## Strengths and limitations

Following the call for a joint investigation of the effects of perceived stress and cortisol levels during pregnancy [16], we applied a unified psychological and biological approach to the study of prenatal stress. Studying both influences at the same time within the same sample allowed us to examine the effect of one while controlling for the effect of the other. In addition, we were able to test a 9-year longitudinal relationship, covering a longer time-period than the majority of studies investigating the association between prenatal stress and child externalizing behavior. A further strength of this study is the investigation of sex-dependent effects of indicators of prenatal stress, a research question which has been neglected in various prior studies. As for the outcome of externalizing behavior, we could draw from self- and maternal reports tapping on temperamental features and conduct problems. This approach also makes the concept of externalizing problems accessible for study in a community sample, where the majority of children will not meet criteria for an externalizing disorder. Despite considerable strengths regarding the methodological approach of our study, there are also some limitations that need to be considered. First, the sample size is relatively small for structural equation modelling and investigating sex as a moderator. The small sample sizes for boys and especially girls result in a substantial uncertainty in parameter estimates. Replication of the reported effects in studies with larger sample size is needed. Secondly, at the time of the first study wave, women were instructed to collect samples in each pregnancy trimester on three consecutive days within a designated two-hour time window. However, awakening time, which influences diurnal cortisol patterns [84] was not assessed at the time and therefore not included in analyses. Moreover, state of the art assessment of basal cortisol now involves multiple samples per day in order to map the diurnal rhythm of cortisol secretion [85]. Additionally, participants collected their samples at home and reported their sampling times manually. Recent technical improvements allow the use of electronic devices to monitor participant adherence to sampling instructions [86]. However, even in studies with multiple daily sampling time points, time-adherent sampling in participants not aware of being monitored overall ranges between 71 and 84% [87–89] and some evidence has shown that indicators of low risk (e.g. higher social support, higher education, non-maltreating families) are associated with higher compliance to cortisol sampling instructions [90–93]. Given our low-risk sample and the fact that participants were given a two-hour time window to collect their samples, we would therefore suspect above-average compliance. Further, even though it is now common to collect several samples per day, single saliva samples collected around the same time of day on consecutive days have been shown to be significantly correlated and are indicative of basal cortisol secretion [48].

## Conclusions and future directions

This longitudinal study shows that prenatal psychological stress and cortisol are associated with child behavior nine years later, stressing the significance of early life influences on the development of externalizing behavior. Perceived stress and cortisol had different relationships with child externalizing behavior, suggesting that there might be other mechanisms accounting for the effect of psychological stress. More studies are needed in order to identify the processes that link perceived stress and cortisol respectively to child externalizing behavior. Given that opposing effects were found for girls vs. boys, our study also shows the importance of considering sex as a moderator of the link between prenatal stress and child mental health and temperamental outcomes. In order to gain greater understanding of the mechanisms that account for effects of prenatal stress, future studies could e.g. examine (a) different biological processes relevant to the intrauterine environment and how they differ for male vs. female fetuses, (b) the possibility of intergenerational transmission of HPA (hypo)activity which could be related to externalizing behavior, and (c) postnatal maternal behavior patterns and child responses to them, and whether they might be sex-specific. Given the risk externalizing behavior poses for the development of certain psychiatric disorders and social maladjustment, the study of its predisposing factors remains an important area of research that can guide preventive measures.

Another question deserving additional research concerns the multi-finality of prenatal stress: Can we—for example—determine under which circumstances it will increase the risk for internalizing vs. externalizing problems? As it has been suggested, offspring sex may be one moderator of this relationship, with prenatal stress increasing fear and affective problems in girls and behavioral problems in boys [35]. Which neurodevelopmental or socializing processes may account for this effect, and whether there might be other moderators determining the outcome quality of exposure to prenatal stress, should be investigated in future studies.

### Supplementary Information

**Additional file 1: Table S1**: Pregnancy perceived stress with absent vs. present medical risk factors during pregnancy (Welch’s test). **Table S2**: Pregnancy cortisol with absent vs. present medical risk factors during pregnancy (Welch’s test).

## Data Availability

The study data are available from the corresponding author upon reasonable request.
