# Supplementary material for: Prenatal stress and child externalizing behavior: effects of maternal perceived stress and cortisol are moderated by child sex
Source: Child Adolesc Psychiatry Ment Health. 2023 Aug 7;17:94. doi: 10.1186/s13034-023-00639-2 (PMC10408175; doi:10.1186/s13034-023-00639-2)
Supplement: Supplementary file 1 — Additional file 1: Table S1: Pregnancy perceived stress with absent vs. present medical risk factors during pregnancy (Welch’s test). Table S2: Pregnancy cortisol with absent vs. present medical risk factors during pregnancy (Welch’s test). [file 13034_2023_639_MOESM1_ESM.pdf]

## Additional file Tables

Table S1: Pregnancy perceived stress with absent vs. present medical risk factors during pregnancy (Welch's test)

| | PSQ Risk absent | | PSQ Risk present | | t | Welch's df | p |
|---|---|---|---|---|---|---|---|
| | *M* | *SD* | *M* | *SD* | | | |
| Pregnancy complications t1 | 38.844 | 20.03 | 42.425 | 18.85 | -.9 | 79.07 | .362 |
| Pregnancy complications t2 | 37.855 | 19.14 | 40.873 | 19.83 | -.75 | 66.09 | .467 |
| Pregnancy complications t3 | 37.596 | 19.93 | 43.536 | 18.50 | -1.55 | 89.48 | .125 |
| Alcohol consumption t1 | 39.596 | 19.71 | 48.148 | 17.56 | -1.15 | 6.07 | .293 |
| Alcohol consumption t2 | 38.882 | 19.52 | 38.334 | 3.92 | .15 | 4.69 | .879 |
| Alcohol consumption t3 | 40.130 | 19.70 | 34.444 | 16.46 | .75 | 4.89 | .490 |
| Hospitalization t1 | 40.50 | 19.95 | 37.89 | 18.64 | .42 | 11.76 | .683 |
| Hospitalization t2 | 38.91 | 19.46 | 38.63 | 19.22 | .05 | 18.26 | .960 |
| Hospitalization t3 | 39.13 | 19.94 | 41.97 | 18.47 | -.68 | 50.14 | .503 |

*Note*: t1 = 1st pregnancy trimester, t2 = 2nd pregnancy trimester, t3 = 3rd pregnancy trimester

Table S2: Pregnancy cortisol with absent vs. present medical risk factors during pregnancy (Welch's test)

| | Cortisol Risk absent | | Cortisol Risk present | | t | Welch's df | p |
|---|---|---|---|---|---|---|---|
| | *M* | *SD* | *M* | *SD* | | | |
| Pregnancy complications t1 | 2.90 | 1.22 | 2.88 | .96 | .08 | 89.13 | .934 |
| Pregnancy complications t2 | 2.81 | 1.12 | 3.02 | 1.05 | -.87 | 72.24 | .389 |
| Pregnancy complications t3 | 2.71 | 1.19 | 3.08 | .97 | -1.70 | 96.86 | .092 |
| Alcohol consumption t1 | 2.89 | 1.15 | 2.86 | .95 | .02 | 4.90 | .988 |
| Alcohol consumption t2 | 2.89 | 1.13 | 2.51 | .86 | .61 | 1.22 | .637 |
| Alcohol consumption t3 | 2.85 | 1.13 | 2.97 | 1.01 | -.19 | 2.31 | .812 |
| Hospitalization t1 | 2.92 | 1.12 | 2.94 | 1.13 | -.07 | 11.43 | .942 |
| Hospitalization t2 | 2.89 | 1.14 | 2.86 | 1.06 | .09 | 17.29 | .927 |
| Hospitalization t3 | 2.82 | 1.17 | 2.95 | .98 | -.55 | 52.95 | .581 |

*Note*: t1 = 1st pregnancy trimester, t2 = 2nd pregnancy trimester, t3 = 3rd pregnancy trimester
